# Community Outbreak of Adenovirus, Taiwan, 2011

**DOI:** 10.3201/eid1811.120629

**Published:** 2012-11

**Authors:** Tsung-Pei Tsou, Boon-Fatt Tan, Hsin-Yu Chang, Wan-Chin Chen, Yuan-Pin Huang, Chen-Yin Lai, Yen-Nan Chao, Sung-Hsi Wei, Min-Nan Hung, Li-Ching Hsu, Chun-Yi Lu, Pei-Lan Shao, Jung-Jung Mu, Luan-Yin Chang, Ming-Tsan Liu, The Unknown Pathogen Discovery/Investigation Group, Li-Min Huang

**Affiliations:** Centers for Disease Control, Taipei, Taiwan (T.P. Tsou, W.C. Chen, Y.P. Huang, Y.N. Chao, S.H. Wei, M.N. Hung, L.C. Hsu, J.J. Mu, M.T. Liu);; and National Taiwan University Hospital, Taipei (B.F. Tan, H.Y. Chang, C.Y. Lai, C.Y. Lu, P.L. Shao, L.Y. Chang, L.M. Huang)

**Keywords:** adenovirus, surveillance, pneumonia, outbreak, viruses, Taiwan, severe infection, adenovirus type 7, HAdV, human adenovirus

## Abstract

In 2011, a large community outbreak of human adenovirus (HAdV) in Taiwan was detected by a nationwide surveillance system. The epidemic lasted from week 11 through week 41 of 2011 (March 14–October 16, 2011). Although HAdV-3 was the predominant strain detected (74%), an abrupt increase in the percentage of infections caused by HAdV-7 occurred, from 0.3% in 2008–2010 to 10% in 2011. Clinical information was collected for 202 inpatients infected with HAdV; 31 (15.2%) had severe infection that required intensive care, and 7 of those patients died. HAdV-7 accounted for 10%, 12%, and 41% of infections among outpatients, inpatients with nonsevere infection, and inpatients with severe infection, respectively (p<0.01). The HAdV-7 strain detected in this outbreak is identical to a strain recently reported in the People’s Republic of China (HAdV7-HZ/SHX/CHN/2009). Absence of circulating HAdV-7 in previous years and introduction of an emerging strain are 2 factors that caused this outbreak.

## Abstract 2

Adenovirus type 7 caused a high proportion of severe infections.

Human adenoviruses (HAdVs) are DNA viruses that can cause a variety of human diseases. Since the discovery of these viruses in 1953, more than 50 types have been isolated, some directly linked to specific human diseases, e.g., infantile diarrhea (HAdV 40, 41), epidemic keratoconjunctivitis (HAdV 8, 19, 37, 54), and hemorrhagic cystitis (HAdV 11, 21) (*1*–*3*). Adenoviruses also are common causes of lower respiratory tract infections in children (*4*), but surveillance for these infections is lacking in most countries. Pneumonia caused by adenoviruses cannot be easily differentiated from other types of viral pneumonia, and culturing and typing of adenoviruses is not routinely performed in hospital or public health laboratories. Therefore, community-wide outbreaks of adenovirus are not easily detected; previous reports are limited to those occurring in hospital, school, or military settings (*5*–*7*).

In 1999, after a 1998 enterovirus 71 epidemic, the Taiwan Centers for Disease Control established a nationwide surveillance system using contract virologic laboratories (CVLs) to perform continuous virologic surveillance for respiratory viruses, especially influenza and enteroviruses (*8*). The network consists of 12 CVLs located in the northern, central, southern, and eastern regions of Taiwan (*9*). Early in 2011, the percentage of adenovirus isolated among all respiratory virus isolates evaluated by the CVLs increased from a baseline of 0%–5% to 10% and remained high in the following weeks, indicating a community-wide adenovirus outbreak. The apparent outbreak prompted us to use several existing surveillance systems to describe the characteristics of this outbreak.

## Methods

### Virologic Surveillance of Outpatients

For respiratory virus surveillance, throat/nasal swabs from outpatients with influenza-like illness (ILI) are collected by sentinel physicians and cultured in the CVL of the corresponding region on a weekly basis. Virus isolates found are then sent to the reference laboratory at the Taiwan Centers for Disease Control for identification, sequencing, and typing. For this study, we reviewed virologic surveillance data from week 1 of 2008 through week 43 of 2011 (January 1, 2008–October 30, 2011). The weekly adenovirus-positive rate is defined as the number of adenoviruses isolated from respiratory tract specimens divided by the number of all specimens submitted from patients with ILI for respiratory virus surveillance in the corresponding week. The mean positive rate in 2008–2010 was used as the baseline adenovirus-positive rate, and the epidemic threshold was defined as the baseline adenovirus-positive rate + 2 SD. The start and end of the epidemic were defined accordingly.

### Clinical Case Surveillance for Inpatients

We conducted a retrospective study of adenovirus-infected children treated as inpatients in the National Taiwan University Hospital (NTUH), a tertiary hospital in northern Taiwan. We enrolled all patients <18 years of age admitted to the NTUH pediatric department from November 1, 2010 (week 44, 2010), through June 30, 2011 (week 26, 2011), who had adenovirus infection identified by virus isolation or PCR from respiratory specimens. All virus isolations and PCRs were performed in the NTUH laboratory, 1 of the 12 CVLs.

All medical records of enrolled inpatients were reviewed. Demographics, medical history, clinical signs and symptoms, diagnoses, and treatments were recorded by using a structured questionnaire. Patients who had been admitted to the intensive care unit were classified as having severe infection; all other patients were classified as having nonsevere infection.

### Virus Culture, Identification, and Typing

We typed selected adenovirus isolates collected as part of the virologic surveillance program and all isolates obtained from inpatients from NTUH. We also performed real-time PCR for viral load quantification by using LightCycler (Roche Diagnostics, Mannheim, Germany) for selected patients from the NTUH, according to the method described by Heim et al. (*10*).

Throat/nasal swab specimens from all patients were injected into human embryonic lung fibroblasts, Hep-2, RD, MK-2, and MDCK cells. If a cytopathic effect was observed, the presence of adenovirus was further confirmed by direct immunofluorescence staining with a virus-specific monoclonal antibody. DNA was extracted from the clinical samples by using the QIAamp Blood Mini Kit (QIAGEN, Hilden, Germany); PCR was then conducted targeting a 956-bp region of the hexon gene for typing.

For genetic analyses of HAdV-7 isolates, DNA fragments of the hexon and fiber genes were amplified by PCR. Multiple sequence alignments, protein translation, and phylogenetic analysis were performed on the basis of the nucleotide sequences by using MEGA4 (*11*) and BioEdit software (www.mbio.ncsu.edu/BioEdit/bioedit.html). For phylogenetic analyses, we included the full-length sequences of the hexon and fiber genes (2,805 bp and 978 bp, respectively) from 5 HAdV-7 isolates collected in 2011 in Taiwan and some reference sequences available in the National Center for Biotechnology Information database (www.ncbi.nlm.nih.gov/genbank). A phylogenetic tree was constructed by the neighbor-joining method, and 1,000 bootstrap replications were performed to evaluate the reliabilities of the relationships.

### Statistical Analysis

Data from inpatients with versus without severe infection were compared by using the χ^2^ or Fisher exact test for categorical variables and the Mann-Whitney U test for continuous variables. A p value <0.05 was considered significant. All statistical operations were 2-tailed and were performed with SPSS version 19.0 software (IBM, Somers, NY, USA).

## Results

### Virologic Surveillance for Outpatients

**Figure 1:**
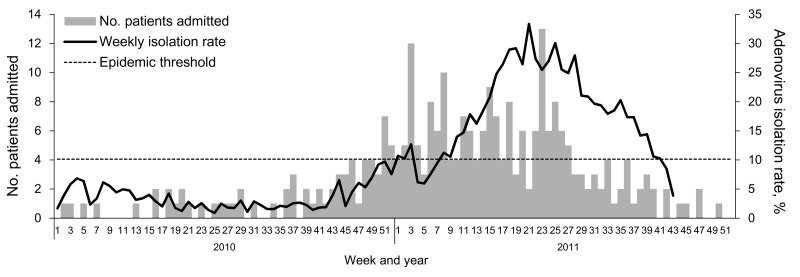
Weekly adenovirus-positive rates for respiratory specimens from patients with influenza-like illness sent to contract virologic laboratories at the Taiwan Centers for Disease Control and weekly number of inpatients infected with adenovirus in the pediatric department of National Taiwan University Hospital, Taipei City, Taiwan, week 1, 2010–week 43, 2011 (January 1, 2010–October 30, 2011). Weekly adenovirus positive rate = no. adenovirus isolates from respiratory tract specimens / no. all specimens submitted to contract virologic laboratories from outpatients with influenza-like illness for respiratory virus surveillance in the corresponding week.

From week 1 of 2008 through week 43 of 2011, an average of 276 respiratory tract specimens from ILI outpatients were collected each week by the CVLs (range 27–1,028, SD 130). Weekly adenovirus-positive rates are shown in Figure 1. The baseline adenovirus-positive rate in 2008–2010 was 5.75% (range 0%–13.1%, SD 3.37%); the epidemic started in week 11 and ended in week 41 of 2011. Mean adenovirus-positive rate during the epidemic was 25.9%, with a peak of 37.3% during week 21 of 2011. Ninety-seven percent of all specimens were collected from patients <18 years of age.

**Figure 2:**
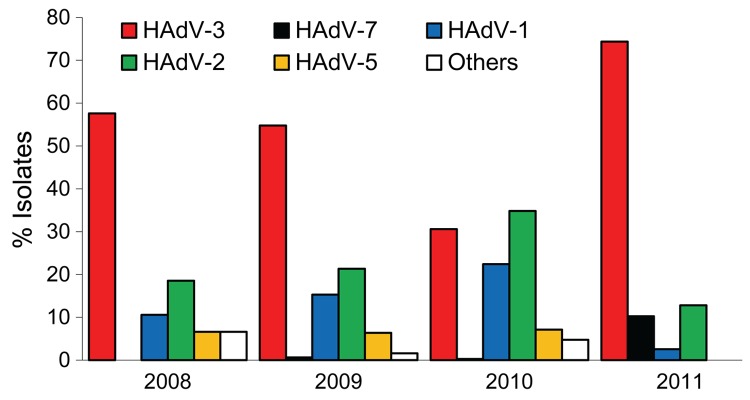
Distribution of adenovirus (HAdV) types in respiratory samples collected from outpatients <18 years of age by contract virologic laboratories in Taiwan, 2008–2011.

We typed 883 adenovirus isolates collected during the study period from outpatients <18 years of age; temporal distribution is shown in Figure 2. HAdV-3 was the dominant circulating type during 2008–2009 (>50% of all isolates), but in 2010, the proportion of HAdV-3 decreased to ≈30%, similar to that for HAdV-2 and HAdV-1. The proportion of HAdV-5 was consistently ≈5% in 2008–2010, and all other types of adenoviruses were rarely isolated. Among the 844 adenovirus isolates collected during 2008–2010 that were typed, only 3 (0.3%) were HAdV-7; during this outbreak, the proportion of HAdV-7 increased significantly, to 10% (p<0.001). The proportion of HAdV-3 also increased, from 30% to 74%, while the proportions of all other types decreased significantly (p<0.001).

### Clinical Case Surveillance for Inpatients

#### Epidemiology and Typing

We enrolled 202 inpatients at the NTUH pediatric department who tested positive for adenovirus from November 1, 2010 (week 44, 2010), through June 30, 2011 (week 26, 2011). All but 3 of these patients had positive test results for adenovirus by culture; the remaining 3 patients had positive test results by PCR only. The number of hospitalized patients increased following week 43 of 2010 and remained high in the following months, a trend that is congruent with virologic surveillance results (Figure 1). Of the 202 patients, 31 had severe infections requiring admission to the intensive care unit and 171 had nonsevere infections.

Hexon gene sequencing showed that HAdV-3 accounted for 64% of all isolates among inpatients; HAdV-7 (19%), HAdV-2 (4%), and HAdV-1 (2%) were the next most prevalent types. HAdV-5, HAdV-6, and HAdV-37 accounted for 2 isolates each (1%). Sixteen isolates (8%) were not typeable because the specimens were inadequate.

The dominant HAdV type differed for patients with versus without severe infection. HAdV-3 infection was more common among patients with nonsevere versus severe infection (68% vs. 42%; p<0.001), whereas HAdV-7 was the dominant type among patients with severe versus nonsevere infection (45% vs. 14%; p<0.001). In the nonsevere infection group, other identified adenovirus types were HAdV-2 (4%), HAdV-1 (2%), HAdV-5 (1%), HAdV-6 (1%), and HAdV-37 (1%). In the severe infection group, only HAdV-2 (10%) and HAdV-6 (3%) were identified in addition to HAdV-7 and HAdV-3.

#### Demographics and Associated Conditions

Demographic data on inpatients is shown in Table 1. Median age was 40 months (range 1–189 months); the male:female ratio was 1.3:1. Most (59%) patients had a history of contact before illness onset with patients with upper respiratory symptoms in school or in the household. Patients with severe infection had longer hospital stays than did those with nonsevere infection (median 18 vs. 5 days; p<0.001). Thirty-eight percent of all patients had chronic underlying conditions before admission, and more patients with severe infection had underlying diseases, particularly cardiopulmonary and neurologic diseases, than did those with nonsevere infection (77% vs. 30%; p<0.001).

**Table 1:** Characteristics of 202 children hospitalized for adenovirus infection at National Taiwan University Hospital, by disease severity, Taipei City, Taiwan, November 2010–June 2011*

Characteristic	All	Severe infection, n = 31	Nonsevere infection, n = 171	p value
Median age, mo (range)	40 (1–189)	36 (1–185)	41 (2–189)	0.287
Male sex	115 (57)	21 (68)	94 (55)	0.186
Contact history	119 (59)	15 (48)	104 (61)	0.196
Median hospitalization, d (range)	5 (1–126)	18 (4–126)	5 (1–44)	<0.001
Death rate	7 (4)	7 (23)	0 (0)	<0.001
Any underlying disease	76 (38)	24 (77)	52 (30)	<0.001
Prematurity	36 (18)	8 (26)	28 (16)	0.207
Cardiopulmonary†	31 (15)	12 (39)	19 (11)	<0.001
Neurologic‡	25 (12)	19 (61)	6 (4)	<0.001
Hematologic§	2 (1)	1 (3)	1 (0.6)	0.284
Metabolic¶	7 (4)	4 (13)	3 (2)	0.012
Immunodeficiency#	1 (0.5)	1 (3)	0 (0)	0.153

*Values are no. (%) patients except as indicated. Severe infection indicates patients who were admitted to the intensive care unit. †Congenital heart diseases (12), asthma (11), chronic lung disease (4), tracheobronchial stenosis (2), and laryngo-tracheo-bronchomalacia (2). ‡Cerebral palsy, epilepsy, and psychomotor retardation. §Acute myeloid leukemia and Burkitt’s leukemia. ¶Pompe disease, metachromatic leukodystrophy, glycogen storage disease, isovaleric acidemia, short-chain acyl-coA dehydrogenase deficiency, renal tubular acidosis, and mitochondrial disease. #Hypogammaglobulinemia.

#### Clinical and Laboratory Characteristics

Clinical and laboratory characteristics of HAdV infections among inpatients are shown in Table 2 and Table 3. Fever (98%), cough (85%), and coryza (84%) were the most common symptoms. Compared with patients who had nonsevere infection, patients with severe infection had a longer fever duration (median 11 vs. 5 days; p<0.001) and a higher peak temperature (median 39.8 vs. 39.3°C; p = 0.008). Patients with severe infection also had more signs and symptoms of lower respiratory tract involvement than did patients with nonsevere infection, e.g., dyspnea (90% vs. 21%; p<0.001), rales (84% vs. 40%; p<0.001), wheezing (61% vs. 18%; p<0.001), and patches or consolidation on a radiograph (71% vs. 19%; p<0.001). In contrast, exudative tonsillitis was more common for patients with nonsevere than with severe infection (41% vs. 13%; p = 0.003). One fourth of all patients had gastrointestinal manifestations, regardless of infection severity.

**Table 2:** Clinical signs and symptoms of 202 children hospitalized for adenovirus infection at National Taiwan University Hospital, by disease severity, Taipei City, Taiwan, November 2010–June 2011*

Signs and symptoms	All	Severe infection, n = 31	Nonsevere infection, n = 171	p value
Fever	198 (98)	31 (100)	167 (98)	1.000
Duration, d (range)	6 (0–28)	11 (1–28)	5 (0–20)	<0.01
Median peak temperature, °C (range)	39.4 (37.0–41.2)	39.8 (38.7–40.7)	39.3 (37.0–41.2)	0.008
Cough	172 (85)	27 (87)	145 (85)	1.000
Coryza	170 (84)	23 (74)	147 (86)	0.111
Dyspnea	64 (32)	28 (90)	36 (21)	<0.01
Abdominal pain	30 (15)	6 (19)	24 (14)	0.419
Vomiting	51 (25)	6 (19)	45 (26)	0.412
Diarrhea	57 (28)	10 (32)	47 (28)	0.587
Sore throat	47 (23)	5 (16)	42 (25)	0.307
Conjunctivitis	35 (17)	2 (7)	33 (19)	0.082
Exudative tonsillitis	74 (37)	4 (13)	70 (41)	0.003
Rash	15 (7)	3 (10)	12 (7)	0.707
Abnormal breath sound				
Rales	95 (47)	26 (84)	68 (40)	<0.01
Wheeze	49 (24)	19 (61)	30 (18)	<0.01
Chest radiograph finding				
Infiltrate	168 (70)	25 (81)	106 (62)	0.045
Patch/consolidation	92 (39)	22 (71)	33 (19)	<0.01
Pleural effusion	6 (3)	3 (10)	3 (2)	0.016

*Values are no. (%) patients except as indicated. Severe infection indicates patients who were admitted to the intensive care unit.

**Table 3:** Laboratory test results for 202 children hospitalized for adenovirus infection at National Taiwan University Hospital, by disease severity, Taipei City, Taiwan, November 2010–June 2011*

Laboratory results and no. patients tested	All	Severe infection, n = 31	Nonsevere infection, n = 171	p value
Hemoglobin, g/dL (range), n = 201	11.7	9.7 (3.4–13.8)	11.8 (7.0–14.6)	<0.001
Platelets, × 10^3^/μL (range), n = 201	237	106 (11–340)	251(14–555)	<0.001
Thrombocytopenia, no. (%) patients†	43 (21)	24 (77)	19 (11)	<0.001
Leukocytes, × 10^3^ cells/μL (range), n = 201	10.06	4.4 (1.2–10.3)	11.3 (0.2–35.5)	<0.001
Leukopenia, no. (%) patients‡	28 (14)	18 (58)	10 (6)	<0.001
Sodium, mmol/L (range), n = 138	135	131 (119–137)	135 (127–142)	<0.001
Hyponatremia, no. (%) patients§	68 (49)	22 (81)	46 (41)	<0.001
C-reactive protein, mg/dL (range), n = 200	4.15	6.1 (0.02–30.00)	4.0 (0–47.2)	0.086
LDH, U/L (range), n = 22	2,202	3,281 (1,029–9,782)	937 (493–17,454)	0.023
AST, U/L (range), n = 117	43	135 (38–3,520)	38 (3.5–989)	<0.001
AST >2× upper limit, no. (%) patients	36 (31)	22 (85)	14 (15)	<0.001
ALT, U/L (range), n = 104	26.5	67.5 (12.0–511.0)	24 (8–652)	<0.001
ALT >2× upper limit, no. (%) patients	24 (23)	15 (58)	9 (12)	<0.001
Creatinine, mg/dL (range), n = 155	0.61	0.68 (0.3–1.1)	0.6 (0.2–0.9)	0.088

*Severe infection indicates patients who were admitted to the intensive care unit. LDH, lactate dehydrogenase; AST, aspartate aminotransferase; ALT, alanine aminotransferase. †Platelet count <150,000/μL. ‡Leukocyte count <5,000 cells/μL. §Serum sodium <135 mmol/L.

Compared with nonsevere infection patients, patients with severe infection had lower hemoglobin levels (9.7 vs. 11.8 mg/dL; p<0.001) and more frequently had leukopenia (leukocyte count <5 × 10^3^ cells/μL; 58% vs. 6%; p<0.001) and thrombocytopenia (platelet count <150 × 10^3^/μL; 77% vs. 11%; p<0.001). Lactate dehydrogenase level was elevated in all patients, but the level was significantly higher among those in the severe infection group (3,281 vs. 937 U/L; p = 0.02). Serum sodium levels tended to be low in all patients, but hyponatremia was significantly more common for patients with severe versus nonsevere infection (81% vs. 41%; p<0.001).

Adenovirus viral load was determined by real-time PCR from throat swabs (n = 26), sputum (n = 5), or pleural effusion (n = 10). Median copy numbers were 2.00 × 10^7^ (range 1.60 × 10^5^–3.00 × 10^9^) for throat swabs, 5.30 × 10^8^ (range 3.10 × 10^5^–9.90 × 10^11^) for sputum, and 7.95 × 10^8^ (range 4.80 × 10^7^–2.40 × 10^10^) for pleural effusion. Viral load did not differ between patients with severe versus nonsevere infection.

#### Diagnosis, Treatment, and Prognosis

The most common clinical diagnosis among patients in this outbreak was pneumonia/bronchopneumonia (31.3%); other notable conditions diagnosed were acute tonsillitis (20.1%), acute sinusitis (9.6%), acute otitis media (9.3%), acute gastroenteritis (5.6%), upper respiratory tract infection (4.4%), pharyngoconjunctival fever (4.0%), acute bronchiolitis/bronchitis (4.0%), encephalitis (1.9%), empyema (0.9%), and croup (0.3%). One child in whom Kawasaki disease was initially diagnosed later was determined to be infected with HAdV-3. HAdV-6 was isolated from the throat of a child in whom acute myocarditis had been diagnosed. Other diagnoses included orbital cellulitis, viral exanthem, acute hepatitis, young infant fever, and febrile convulsions.

Parenteral or oral antimicrobial drugs were given to 73.8% of patients before adenovirus infection was diagnosed. Ten percent of patients required mechanical ventilation during hospitalization; all were in the severe group. Intravenous immunoglobulin (IVIG) was given to 6 patients (3.0%) for diagnoses of acute myocarditis (n = 1), empyema (n = 1), acute respiratory distress syndrome (n = 3), and Kawasaki disease (n = 1). Extracorporeal membrane oxygenation was used for 5 patients with respiratory or cardiovascular failure; 3 survived.

Seven patients died during this outbreak: 4 patients infected with HAdV-7, 2 with HAdV-3, and 1 with HAdV-2. All of these patients were in the severe group and had underlying diseases; 6 were bedridden before hospital admission. During hospitalization, all of these patients had secondary bacterial pneumonia develop; pathogens involved were *Pseudomonas aeruginosa*, *Staphylococcus aureus*, *Acinetobacter baumannii*, and *Escherichia coli*.

### Molecular Studies on Adenovirus Type 7

**Figure 3:**
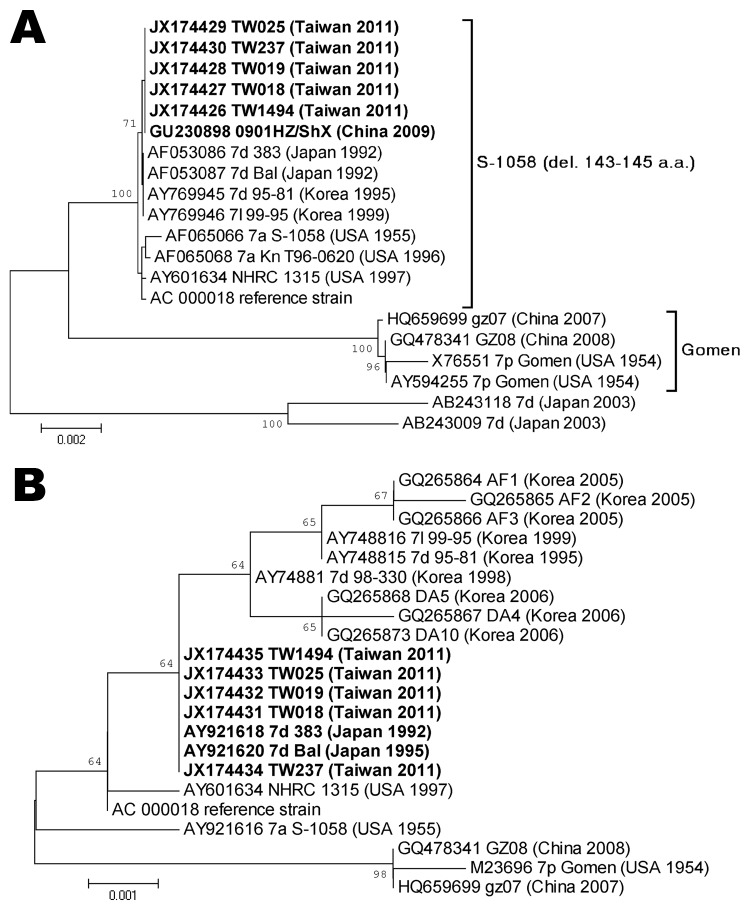
Phylogenetic analysis of hexon (A) and fiber (B) genes of human adenovirus (HAdV) type 7 isolates. Coding sequences of hexon and fiber genes (2,805 and 978 bp) from 5 HAdV isolates from Taiwan in 2011 and reference sequences from the National Center for Biotechnology Information (www.ncbi.nlm.nih.gov/genbank) were included. Phylogenetic trees were constructed from aligned sequences by using the neighbor-joining method; 1,000 bootstrap replications were performed to evaluate the reliabilities. Bootstrap values are shown at branching points. Taxon names include accession number, available genotype, strain name, isolation country, and year. **Boldface** indicates the isolates collected in Taiwan and reference isolates with identical sequences. TW1494 is from an outpatient; TW018, 019, 025, and 237 are from inpatients. del., deletion. Scale bars indicate nucleotide substitutions per site.

The hexon gene sequences of HAdV-7 isolates from virologic surveillance and clinical case surveillance, including patients with severe and nonsevere infection, were determined and compared with strains isolated previously in Taiwan and other countries. Hexon nucleotide sequences of 5 HAdV-7 isolates collected in 2011 in Taiwan were identical to the sequence of strain HAdV7-HZ/SHX/CHN/2009 (*6*,*12*), differed by 1 bp of synonymous mutation from genotype 7d of the 383 strain isolated in Japan in 1992, and had 99.7% identity to the sequence of the prototype strain S1058 (Figure 3, panel A). Fiber sequences of these isolates were identical to those of the 383 and Bal strains from Japan, isolated in 1992 and 1995, respectively, and had 99.5% identity to the prototype stain S1058 (Figure 3, panel B). We found no substantial differences between the HAdV-7 isolates collected from outpatients as part of the virologic surveillance program and the isolates from inpatients with severe or nonsevere infection.

## Discussion

Adenoviruses circulate year-round in Taiwan, and several community outbreaks have been reported. During 1999–2001, outbreaks were caused, consecutively, by HAdV-7, HAdV-3, and HAdV-4 (*13*); another HAdV-3 outbreak was reported during 2004–2005 (*14*). For these outbreaks, the adenovirus-positive rate increased from 7% during the baseline period to 14%–16% during the outbreak.

The outbreak of co-circulating HAdV-3 and HAdV-7 we report is unique in several ways. The high positive rate during the weeks of the epidemic made this outbreak among the largest reported community-wide adenovirus outbreaks, and the reemergence of HAdV-7 in Taiwan 10 years after the last outbreak contributed to severe infection.

HAdV-3 and HAdV-7 are both capable of causing outbreaks but have different circulation patterns. HAdV-3 circulates endemically and causes outbreaks, whereas HAdV-7 is mainly detected during outbreaks (*15*,*16*). DNA restriction analysis has been used to further characterize adenoviruses, and shifts in or replacement of the predominant genome types over time have been reported to be related to outbreaks (*17*,*18*). For a novel genome type of HAdV-7 to spread rapidly and cause a large outbreak, an immunologically naive population and a means of introduction to the susceptible community are needed, and the novel virus must have a greater biologic fitness than that of other circulating strains (*18*). During the 1999 outbreak in Taiwan, 40% of adenovirus isolates were HAdV-7. The proportion of HAdV-7 among all adenoviruses decreased to 20% in 2000, <5% in 2001, and <1% in 2004–2005 and 2008–2010 (*13*,*14*). The near-absence of circulating HAdV-7 in 2001–2010 indicates that a large population was naive to this virus, predisposing that population to the 2011 outbreak.

Genome typing showed that HAdV-7a was the circulating strain in Taiwan in 1983 but was replaced by HAdV-7b by 1999–2001 (*13*). However, phylogenetic analysis of the entire hexon gene showed that the strain from this outbreak has the highest homology with HAdV-7d and HAdV-7d2, which had not reported in Taiwan (*6*). HAdV-7d has been the predominant circulating virus in China since the early 1980s and caused a large outbreak in South Korea during 1995–1997 (*16*). The closely related genotype HAdV-7d2 was reported in Israel in 1992 and has caused outbreaks in chronic care facilities and in communities in several countries (*6*,*19*–*21*). Despite the early introduction of HAdV-7d to neighboring countries, HAdV-7d had not been found in Taiwan until 2011 (*13*).

We sequenced the entire fiber gene, the product of which contributes to virus attachment and infection, to further characterize the circulating strain (*22*). Phylogenetic analysis (Figure 3) showed no substantial genome difference between this HAdV-7d strain and other previously reported HAdV-7d strains. We do not know the means of introduction of this strain into Taiwan, but we believe that an emerging strain and the large, naive population are 2 factors that contributed to this outbreak.

HAdV-7 has been reported to have a strong association with severe illness (*23*). Studies of outbreaks caused by the co-circulation of HAdV-3 and HAdV-7 offer a unique opportunity for us to compare the disease severity resulting from infection with these 2 virus types, but conflicting results have been obtained (*7*,*13*,*24*). During this outbreak, although HAdV-3 was the predominant strain, patients infected with HAdV-7 exhibited more severe disease. HAdV-3 accounted for 74%, 62%, and 41% of all isolates in outpatients, inpatients with nonsevere infection, and inpatients with severe infection, respectively, compared with 10%, 12%, and 41%, respectively, for HAdV-7 (p<0.01). Our study offers strong evidence that HAdV-7 is significantly associated with severe infection in a community outbreak setting.

Of the 202 inpatients we analyzed, 31 (15%) had severe infection that required intensive care. As previously reported, children with underlying conditions were more likely to have severe disease develop, especially patients with neurologic, respiratory, and metabolic abnormalities (*23*,*25*–*27*). More than half of inpatients had a history of household or school contact with persons with upper respiratory symptoms before admission, which is an evidence of the high transmissibility of the virus and the widespread nature of the current outbreak.

Inpatients with severe infection had higher fever, longer fever duration, and more evident lower respiratory tract involvement than those with nonsevere infection. Viremia can be found in up to 72% of immunocompetent children during first infection with HAdV (*28*); our laboratory data revealed that adenovirus-infected patients had more profound systemic involvement than was reflected by clinical signs and symptoms, which may have resulted from viremia. Although renal function remained relatively good, other manifestations (e.g., anemia, leukopenia, thrombocytopenia, elevated serum transaminase and lactate dehydrogenase levels) were indicators of multiorgan involvement. Eighty percent of patients with severe infection had hyponatremia, which suggests a syndrome involving inappropriate antidiuretic hormone (*29*). Viral load in respiratory tract specimens did not differ for patients with severe infection versus nonsevere infection, which suggests host factors may be contributors to severe illness.

Compared with that for other reported outbreaks, the mortality rate we found was relatively low (3.5% for all hospitalized patients) (*5*). Good supportive/intensive care and advanced life support, including the use of extracorporeal membrane oxygenation, are possible explanations. With no proven treatment, various medications, including ribavirin (*30*), cidofovir (*31*) and vidarabine (*32*), and IVIG (*33*), have been used to treat immunocompromised patients who have severe adenovirus infection; results have been inconsistent. Six patients in our study received IVIG and 4 of them died, possibly because of severe underlying conditions. We are not able to draw any conclusions regarding the treatment efficacy of IVIG from this small case series; further study is needed.

In conclusion, by using data from public health and hospital-based surveillance programs, we described a large community outbreak caused by circulating HAdV-3 and emerging HAdV-7. We confirmed the severity of HAdV-7 infection and illustrated the epidemic nature of its circulation. Public health surveillance systems should continue to monitor the molecular epidemiology of adenoviruses to detect outbreaks early.
